# Microplastics in the Hamburg port area—an analysis of sediment depth profiles along the upper Elbe river, Germany

**DOI:** 10.1007/s11356-025-35972-w

**Published:** 2025-02-01

**Authors:** Larissa Motyl, Elke Kerstin Fischer

**Affiliations:** Microplastic Research at CEN (MRC, Center for Earth System Research and Sustainability), Universität Hamburg, Bundesstrasse 55, 20146 Hamburg, Germany

**Keywords:** Harbour, Container terminal, Raman spectroscopy, Microbeads

## Abstract

Harbours pose a unique environment of increased anthropogenic pressure and artificial river morphology that are of specific interest concerning microplastic release and accumulation. To address the specific situation in the Hamburg port area, a study in cooperation with the Hamburg Port Authority (HPA) was conducted. Sediment samples at different depth levels were taken at seven sites with similar flow velocity and underlying morphology. Two sites are located upstream and downstream of the port of Hamburg, while four sites are located in the centre area of the port. One additional site takes into account an estuary of a receiving stream flowing into the upper river Elbe. For the analysis of microplastic concentrations in the samples, the biogenic organic matter was removed by oxidative digestion followed by wet sieving and density separation. For identification, the Nile red staining method in combination with fluorescence microscopy was applied. A subset of identified synthetic polymers was investigated for polymer composition via µRaman spectroscopy. In addition, sediment parameters such as grain size distribution, organic matter and water content were analysed. In total, a number of 31 sediment samples divided into different depths below riverbed level were examined. In brief, 11,280 microplastic particles could be identified. Both the highest and lowest number of particles were detected at centre port sites ranging from 60 to 21,799 microplastics per kilogramme dry weight. Fragments are the dominating particle morphology throughout all locations, except for one centre harbour site where microbeads are most common. Frequently appearing synthetic polymers were detected to be polyvinyl chloride (34%) and polyethylene terephthalate (28%). Within this study, a significant correlation between microbead concentrations and the percentage of sand fractions (coarse, middle and fine sand) was detected.

**Supplementary Information:**

The online version contains supplementary material available at 10.1007/s11356-025-35972-w.

## Introduction

The demand for plastic is rising due to its variety of applications and its suitability and durability under different thermal, chemical and biological conditions (Barboza et al. 2019; Stöfen-O’Brien 2015). The largest area of application is disposable packaging production, which accounts for 40% of total plastic consumption in Europe (Worm et al. 2017). Their extreme durability poses an ecological problem as plastics cannot be biodegraded and remain in the environment for a long period of time (Barboza et al. 2019). Especially problematic in this context is the disposal of plastics and their eventual release into the environment. In a study from Borrelle et al. (2020), it is predicted that plastic waste entering aquatic ecosystems could reach 90 Mt/year by 2030. The main reasons for this mismanagement are considered to be improper industrial discharges, insufficient waste management and terrestrial runoff (Claessens et al. 2011; Klein et al. 2015).

Rivers carry between approximately 80% of plastic pollution into the seas with an annual input of an estimated 1.15 and 2.41 million metric tonnes globally (Magyar et al. 2023. This results in an increasing quantity of plastic waste in oceans, seas, lakes and rivers worldwide (Claessens et al. 2011, Strokal et al. 2023). Although the plastic waste that enters these environments is meso-scale in size (centimetres to metres), the majority of plastic that eventually accumulates is much smaller in size (Torrance et al. 2021). Through biological processes, chemical weathering, the physical strength of waves, wind or sand friction as well as UV radiation, heat and oxidation processes, plastic particles degrade and are fragmented into microplastics (Andrady 2011, Campanale et al. 2020, Hernandez et al. 2017, Mathalon and Hill 2014). Microplastics refer to plastic particles up to < 5 mm in diameter (Arthur et al. 2009). Furthermore, microplastics are divided into primary and secondary microplastics according to their generation. The former is industrially produced particles, usually in the morphology of microbeads and pellets used for cosmetic products such as facial exfoliating cleansers, as air-jet boat cleaning media (Mathalon and Hill 2014), as abrasive beads for industrial applications (Nizzetto et al. 2016) or as ion-exchange resins in water treatment plants (Scherer et al. 2020). Secondary microplastics are formed indirectly from the degradation of larger plastic pieces such as fibres and fragments (Blair et al. 2019).

In river territories, harbours pose a unique environment of increased anthropogenic pressure and artificial river morphology that are of specific interest concerning microplastic release and accumulation, the latter especially supported due to reduced flow velocity conditions within the harbour estuary and sub-basins.

The exact sinking processes of microplastics are still largely unknown. Generally, from the density ratio of the plastics and the surrounding water, particles either have a positive or negative buoyancy in water. Particles with a higher density than water therefore sink to the bottom sediment, but also low-density microplastics can be found in large numbers (Choong et al. 2021; Deng et al. 2020; Eo et al. 2019; Klein et al. 2015; Laursen et al. 2022; Scherer et al. 2020; Thompson et al. 2004; Vianello et al. 2013; Zhou et al. 2021). Possible explanation for this phenomenon may be the influence of morphological particle features but also biofilm formation that increase the density of the particles (Mathalon and Hill 2014, Matsuguma et al. 2017, Nizzetto et al. 2016, Qian et al. 2024).

To address the specific situation in the Hamburg port area, a study integrating a spatial analysis was conducted in cooperation with the Hamburg Port Authority (HPA). At seven locations (Oortkaten, Reiherstieg Vorhafen, Hansahafen, Köhlbrand, Parkhafen, Außen-Este and Yachthafen Wedel), sediment depth layers were taken and analysed for microplastic content.

The following study is supposed to give a further understanding about microplastic accumulation within the upper tidal Elbe river addressing the following research questions: (1) are there spatial differences concerning the microplastic concentrations and particle characteristics of sediments within the harbour area, (2) is there a distinct microplastic concentration pattern within sediment depth profiles in the harbour area and (3) are there any correlations between microplastics and sediment parameters.

Since microplastic analysis in harbour areas is still quite rare and difficult to conduct due to their highly variable environment, the following study is supposed to raise awareness of the importance of microplastic research in these highly industrialised areas. Harbour sediments contain complex mixtures of natural and anthropogenic materials, which poses a challenge during particle isolation especially for particles in smaller size fractions (Miller et al. 2022). Another point is that long-term ecological impacts on benthic organisms in harbour sediments as well as the wider food web are still poorly researched. The importance of sediment as a route of microplastic subjection is essential for further research (Sandgaard et al. 2023).

## Material and methods

### Study area and sampling

The Elbe river has its source in Labská louka (Czech Republic) and crosses the Czech Republic, northern and central Germany before discharging into the North Sea near Cuxhaven. The total length of the river amounts to 1091 km, of which 727 km are located in Germany and 364 km in the Czech Republic (Netzband et al. 2002). The Elbe is the third largest river in Central Europe after the Danube and the Rhine based on size, length and catchment area. Approximately 25 million people live in the Elbe catchment in Germany (31% of the total population) and approximately 6 million people in the Czech Republic (58% of the total population). Important tributaries are the Moldau/Vltava, Havel and Saale, which each comprise a catchment area of roughly 25,000 km^2^ of the total Elbe catchment (148,268 km²) (Netzband et al. 2002).

During the Weichselian ice age, the glacial valley of the Elbe functioned as a runoff path for meltwaters. The Elbe valley floor was formed by accumulation processes associated with the late Weichselian to Holocene sea level rise and subsequent advance of tidally controlled peri-marine processes into the Elbe estuary (Ratter 1999). This specific section of the Elbe which is directly influenced by these processes is called the tidal Elbe (Engel and Tode 2007). Starting at the Kugelbake in Cuxhaven, the tidal influence prevails until Geesthacht, where a weir built in 1960 artificially limits the tidal inflow for the rest of the river. It was built to allow better navigability to Lauenburg and to the Elbe-Seitenkanal (Elbe side canal) (Gaumert 1995).

The most important German port cities located at the Elbe river are Dresden, Wittenberg, Meissen and Hamburg (Engel and Tode 2007). The Port of Hamburg is the third largest port in Europe and the largest seaport in Germany (Hochfeld and Röper 2019). It is located 141 km from the North Sea coast and is thus subject to tidal influence. There are no barriers between the Elbe river and the port basins and the daily tidal range affects shipping and port activities (Ratter 1999).

**Fig. 1:**
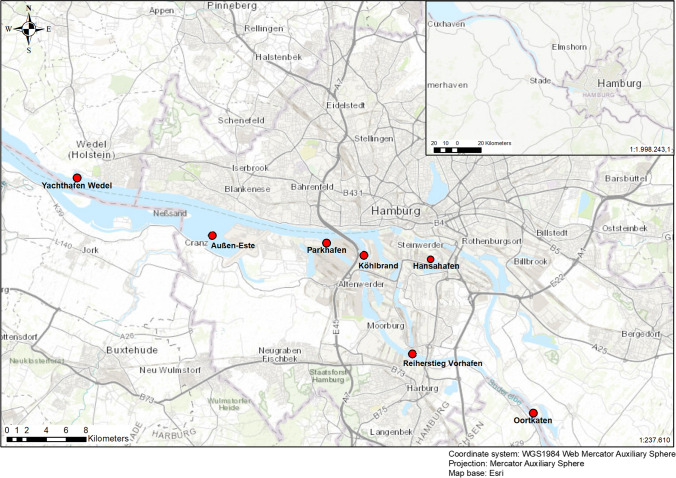
Study area at the Elbe river within the wider area of the port of Hamburg. Red points mark the sampling sites

For the investigation of microplastic concentrations in harbour sediments, seven sediment cores at the sampling sites Oortkaten, Reiherstieg Vorhafen, Hansahafen, Köhlbrand, Parkhafen, Außen-Este and Yachthafen Wedel were taken in cooperation with the Hamburg Port Authority (HPA). The sampling sites Oortkaten, Reiherstieg Vorhafen, Hansahafen, Parkhafen and Yachthafen Wedel were selected based on their similar low flow velocity and sedimentation rates to analyse possible deposition trends of microplastic within the river. These also are influenced by sediment inputs via the upper stream basin and tidal pumping from downstreams. The locations Köhlbrand and Außen-Este were chosen for scientific interest based on their unique localisation within the river and their proximity to the wastewater treatment facilities (Fig. 1).

Each sampling site has specific characteristics in terms of potential microplastic sources. The most upstream site, Oortkaten, is located near a jetty with a few private boats lining the riverbank. A little further downstream lies the sampling site Reiherstieg Vorhafen, which is lined with logistic industries around the riverbank. Further north, the site Hansahafen is located near a pier whereas the site Köhlbrand is located in the downstream part of the Süderelbe directly in the centre of the Port of Hamburg near the Köhlbrand bridge. Even further downstream, Parkhafen is located near a big container terminal. The sampling site Außen-Este is located at the Este estuary, which is a tributary flowing into the upper Elbe. The sampling site is lined with the Airbus shipyard facilities along the riverbank. The last and most downstream site located is Yachthafen Wedel, in the area shortly after the confluence of the Norder- and Süderelbe; the sampling took place directly in a yacht marina.

The sampling campaign of the harbour areas was conducted on the 25^th^, 28^th^ and 29^th^ of September 2020. Samples at all sites were collected at low tide from a boat using a Frahm sediment corer of 15 cm in diameter, provided by the HPA. For the sampling location Außen-Este, a smaller Frahm sediment sampler model with a circumference of 10 cm in diameter was used.

At each site, depth levels were extracted up to 50–90 cm below ground level (bgl). Cores were divided by 10–30 cm according to horizon lines on site. For the first 0–2 cm bgl samples, the upper soil depth level was carefully extracted using a stainless-steel spoon and placed into amber glass jars. The rest of the soil depth levels were placed into 2-l glass jars for transportation to the laboratory. All materials were cleaned thoroughly with MilliQ water, and field blanks were taken for each location to detect the contamination on site. A total number of 31 sediment samples were collected.

Additionally, water quality parameters and flow velocity were conducted at each location using a CTD water probe (conductivity-temperature-depth, Cyclops-7 Turner Designs).

### Laboratory analysis

**Fig. 2:**
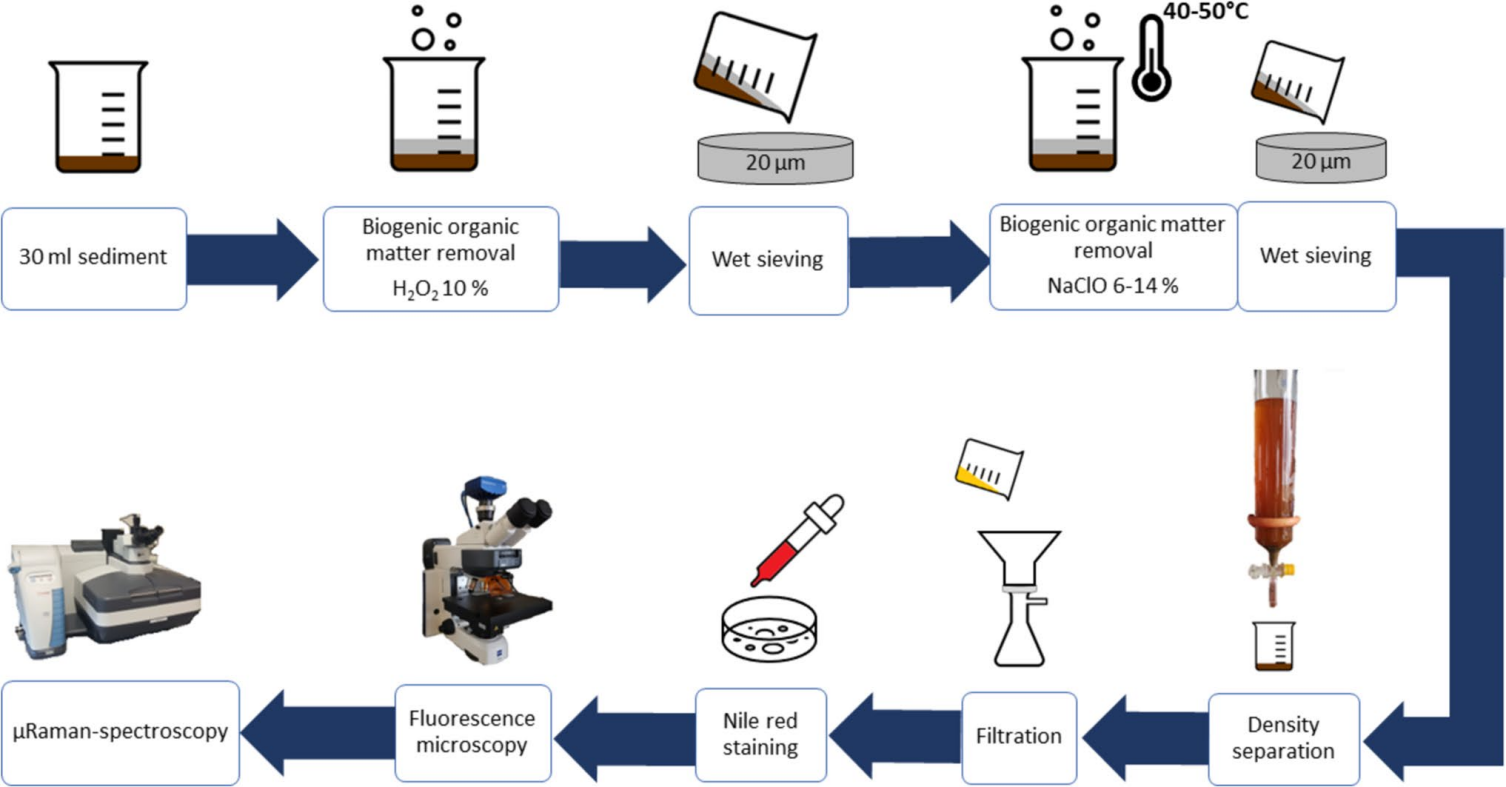
Flowchart of the laboratory analysis

Laboratory analyses were done in the MRC lab (University of Hamburg). Initially, sediment samples were homogenised within the glass jars by thoroughly stirring them with a stainless-steel rod. Subsequently, approximately 30 ml of sediment was transferred into glass beakers and underwent further sample processing (Fig. 2).

#### Digestion of biogenic organic matter

Biogenic organic matter digestion followed a two-step approach. First, 60 ml of hydrogen peroxide (H_2_O_2_, 10%) was added to the samples (exposure time 7 days at room temperature). After removal of the H_2_O_2_ via wet sieving through a stainless-steel sieve with a mesh size of 20 µm, sodium hypochlorite (NaClO, 6–14%) was added to the samples in a volume ratio of 3:1 (Collard et al. 2015). The beakers were placed onto sand baths at 40–50 °C for 24 h.

#### Density separation

To separate microplastic particles from the remaining sediment, a density separation was conducted. For this step, the sample was poured into a 1-l glass column and filled with 700 ml sodium iodide (NaI) solution (density 1.5–1.6 g cm^3^) and sealed at the top with a glass stopper. To eliminate larger particles and to avoid clogging of the glass column, a 1-mm stainless-steel sieve was used prior to pouring the sediment into the column. Residues > 1 mm were inspected visually for potential microplastics. The column was shaken 12 times overhead in a standardised manner to homogenise the sample with the NaI solution. After a sedimentation time of 10 min, the settled sediment on the bottom of the column was discharged through an outlet valve on the column and poured into a glass beaker. The supernatant (approx. 350 ml) was filtered onto paper filters (VWR, qualitative filter paper 413, 5–13 µm particle retention) via a stainless-steel filtration device (Sartorius Combisart), and filters were thoroughly rinsed with MilliQ water. The density separation procedure was repeated three times per sample. At the third round of density separation, the supernatant from the remaining soil suspension was also filtered and transferred onto paper filters. The filters were placed in Petri dishes, were covered with watch glasses and left to dry at room temperature.

#### Nile red staining, fluorescence microscopy and µRaman spectroscopy

For the identification of microplastic particles, the staining approach based on the lipophilic dye Nile red (9-diethylamino-5H-benzo[α]phenoxazin-5-one), according to Tamminga et al. (2017), was applied. Therefore, 1 ml of a solution containing Nile red at a concentration of 1 mg/ml dissolved in chloroform (99% AnalaR Normapur) was added onto the dried paper filters and left to dry.

The filters were then analysed via fluorescence microscopy (AxioScope KMAT 5/7, Zeiss, TRITC Filterset, 2.5x). Particles identified as microplastics were measured for their dimensions and classified according to their morphology as fragments, fibres and microbeads (spherical).

A subset of identified microplastic particles were manually transferred on microscopic slides and analysed for their chemical composition via µRaman spectroscopy (DXR2xi, Thermo Fisher Scientific, laser power 7–10 mW, exposure time 25–10 Hz, 1000 scans, 25-µm confocal pinhole). Resulting spectra were compared to a total of 18 libraries (match at least 70% with additional expert-based evaluation).

Regarding the identification process, the Nile red staining method and fluorescence microscopy proved to be a dependable method. Fischer (2019) demonstrated that 93.4% of particles identified as MP via Nile red staining according to the implemented setup were correctly identified as synthetic polymers by µRaman spectroscopy. Within this study, all identified polymers by fluorescence microscopy were confirmed to be MPs. In terms of accuracy, about 95% MP particles that were priorly pre-selected via Nile red were identified as synthetic polymer. The analysis of false negatives reveals that Nile red staining is underestimating cellulose-based particles and fibres of dark colour.

#### Determination of dry weight, organic matter and grain size composition

To determine the dry weight and water content of the sediment samples, a homogenised volume of 15 ml of sample was dried at 40 °C and subsequently at 105 °C for at least 24 h in a drying oven (DIN ISO 11465). The weight of the sample before and after each drying temperature was recorded with a scale (Sartorius CPA124S, accuracy 0.01 mg). The content of organic matter was determined by annealing approximately 5 g of the samples in the muffle furnace for 4 h at 430 °C and subsequent weighing (DIN EN 12879). In accordance with DIN ISO 11277, the grain size distribution of the samples was analysed.

### Background contamination and procedural blank analysis

Combined field and procedural blanks were taken during the sampling and processing for every sample site. For field blanks, amber glass jars were opened during the sampling, held at a high location and closed when the sample was filled into glass jars and stored for laboratory transport. In the laboratory, blanks were treated the same way as the other sediment samples. Sample series were processed according to site order. The sum of the microplastic particles in the blanks was finally subtracted from the sample values. No microbeads were found in the blank samples. Precautions have been taken in order to minimise background contamination. Therefore, all chemical solutions and MilliQ water were filtered with glass fibre filters (691, VWR International, 1.6-µm retention), and pre-rinsed glass materials were used throughout the processing. Beakers were covered during standing hours, and cotton laboratory coats were worn throughout the processing.

### Statistical analysis

Statistical analyses were performed using the R programming language (version 4.1.2, R Core Team 2022) in the RStudio user interface (version 2021.09.2 + 382, RStudio Team 2021). The geographic information system software ArcGIS (version 10.3.1, Esri) was used to visualise the geospatial data. Concentrations of microplastics were calculated as the number of particles per kilogramme of dry sediment with a correction factor (*K*):

$$K=\frac{1000}{\text{sample dry weight}\, (\text{g})}$$

The Shapiro–Wilk normality test (*α* = 0.05) was performed and did not show a normal distribution (*p* > 0.05) for microplastic concentrations. According to the results, tests for differences in means were performed using the Kruskal–Wallis (*α* = 0.05) test followed by correlation analyses according to SPEARMAN (*α* = 0.05) using IBM SPSS Statistics (IBM Corp. 2019, version 26.0).

## Results

### Procedural blanks

A total number of 4 microplastic fibres and fragments could be detected on blank filters (*n* = 7). The highest contamination was found on the blank for the location Parkhafen (*n* = 3). Majority of particles were identified as fibres.

### Microplastic abundance

**Fig. 3:**
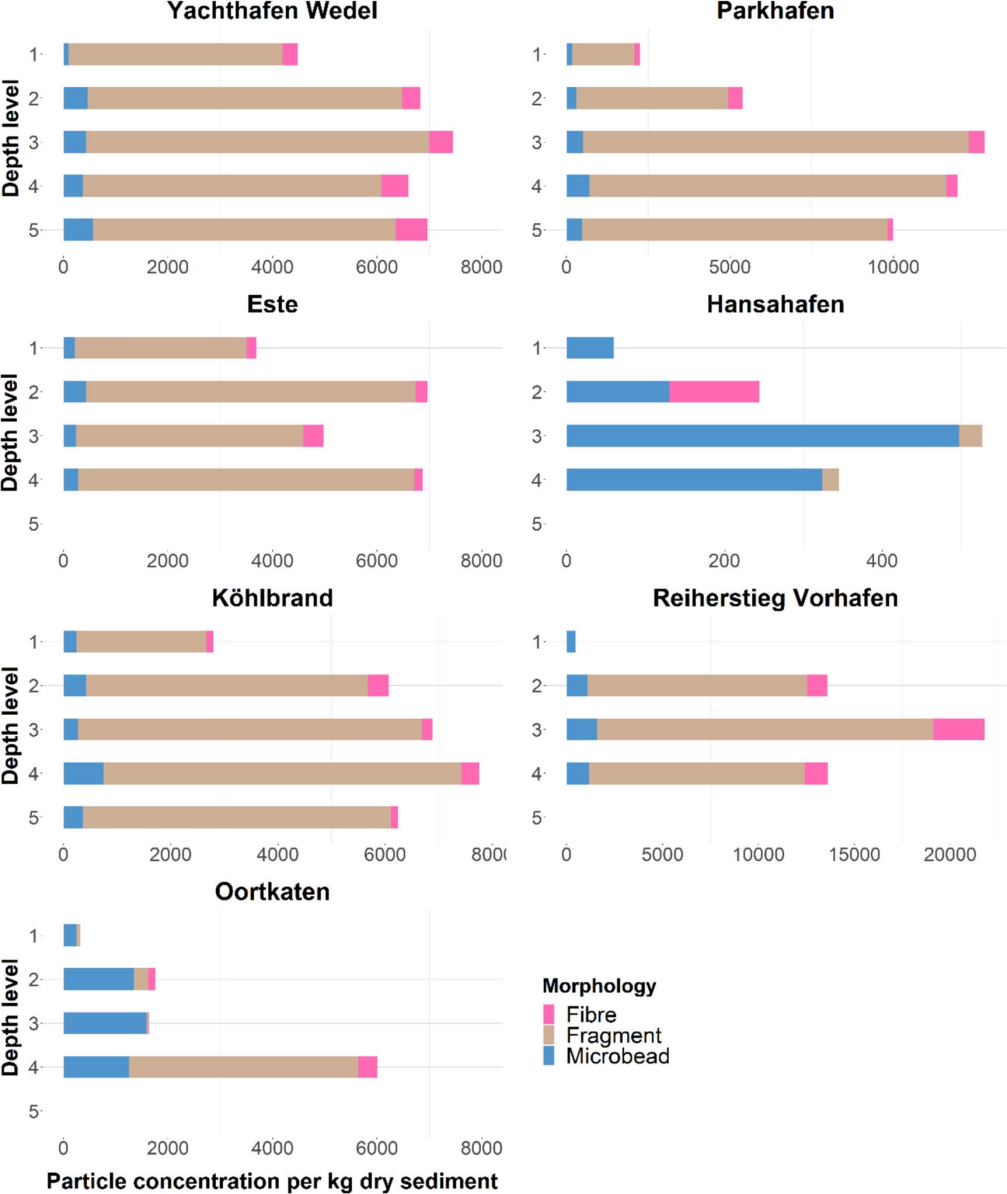
Particle concentration per kg dw according to morphology and locations (different *x*-axis scales at Hansahafen, Parkhafen and Reiherstieg Vorhafen). Please note that depth levels vary according to sampling locations, and level 1 refers to the sediment surface layer

Microplastics could be detected in all of the 31 samples analysed. In total, a number of 11,280 microplastic particles were detected. For each site, the microplastic concentrations were converted into particles per kilogramme dry weight. As seen in Table 1 and Fig. 3, distributions of the locations and depths differ greatly. Depth levels differ at the different locations, since the sampling in an active waterway posed a challenge in sampling and the conditions did not allow a harmonised sediment horizon differentiation for each depth layer. Depths for each level are found in the supporting information (SI5). Interestingly, the first depth levels (surface sediment layer) at all sites showed the lowest microplastic concentration. According to sampling sites, the highest concentration of microplastics was found at Reiherstieg Vorhafen (473–21,799 particles per kg dw). This site also displayed the highest level of contamination at depth level three (20–40 cm depth bgl). In contrast, the lowest number of particles found was at Hansahafen (60–526 particles per kg dw). The complete data are seen in SI5.

**Table 1:** Particle concentrations per kg dw at the first depth level according to morphology and location

Location	Fragments	Microbeads	Fibres	Total particles
Oortkaten	63	254	0	317
Reiherstieg Vorhafen	0	473	0	473
Köhlbrand	2424	239	129	2791
Hansahafen	0	60	0	60
Parkhafen	1903	168	168	2239
Außen-Este	3289	212	186	3687
Yachthafen Wedel	4093	98	294	4485

#### Particle morphologies and size distribution

Fragments were the most common morphology at all locations (85%), followed by microbeads (9%) and fibres (6%) except for the location Hansahafen where the most frequent particle morphology was microbead (as seen in SI1). Another location with a high microbead concentration is Oortkaten, where the first three depth levels showed microbeads as the most dominant type. For the size distribution, four size classes were distinguished ranging from the detection limit of 20 to > 300 µm. The overall size distribution of fragments reveals a median length of 56 µm and a range from 20 up to 2126 µm. Eighty-two percent of all fragments are assigned to the first size class from 20 to 100 µm (Table 2). Microbeads are found predominantly in that same size class (87%), with a median length of 44 µm and ranging from 20 to 617 µm, mostly appearing in the size class > 300 µm to 5 mm with 68%. They have a median length of 525 µm and range from 37 to 4899 µm.

**Table 2:** Size distribution within particle morphologies (%) across all depths

	Size class 1 (20–100 µm)	Size class 2 (100–200 µm)	Size class 3 (200–300 µm)	Size class 4 (> 300 µm)
Fragment	82	16	2	1
Microbead	87	11	2	0.4
Fibre	4	16	11	68

The size distributions according to locations and morphologies are seen in Table 3. The location Außen-Este has the highest median fragment size (80 µm) across all locations. For microbeads, the highest median particle size can be found also at the location Außen-Este with 60 µm. The lowest sizes can be seen at the location Hansahafen for fragments (median 46 µm) and microbeads (36 µm). Fibres reach the highest median value at Yachthafen Wedel (679 µm) and the lowest at Parkhafen (323 µm).

**Table 3:** Descriptive statistics of the size distributions by location and shape

Location	Shape	Min	Max	Median	Mean	sd	1st quart.	3rd quart.
Oortkaten	**Fragment**	21	682	52	63	49	37	75
	**Microbead**	20	256	43	66	49	31	87
	**Fibre**	37	3030	498	781	780	223	1076
Reiherstieg Vorhafen	**Fragment**	20	2007	51	67	65	38	77
	**Microbead**	23	411	41	54	41	32	57
	**Fibre**	50	4899	517	900	985	231	1201
Köhlbrand	**Fragment**	20	2126	58	75	77	43	86
	**Microbead**	23	215	47	52	27	36	60
	**Fibre**	68	4749	538	806	851	259	892
Hansahafen	**Fragment**	26	176	46	73	62	30	130
	**Microbead**	24	617	36	63	84	29	58
	**Fibre**	392	3458	574	1346	1157	531	2089
Parkhafen	**Fragment**	20	583	53	67	48	40	77
	**Microbead**	26	165	44	53	28	36	58
	**Fibre**	57	4235	323	678	890	152	648
Außen-Este	**Fragment**	22	1330	80	99	78	55	119
	**Microbead**	27	424	60	76	65	40	81
	**Fibre**	39	2836	548	872	747	338	1233
Yachthafen Wedel	**Fragment**	20	1449	55	70	58	41	82
	**Microbead**	23	293	53	67	46	40	71
	**Fibre**	41	4874	679	1116	1100	327	1623

#### Polymer composition

**Fig. 4:**
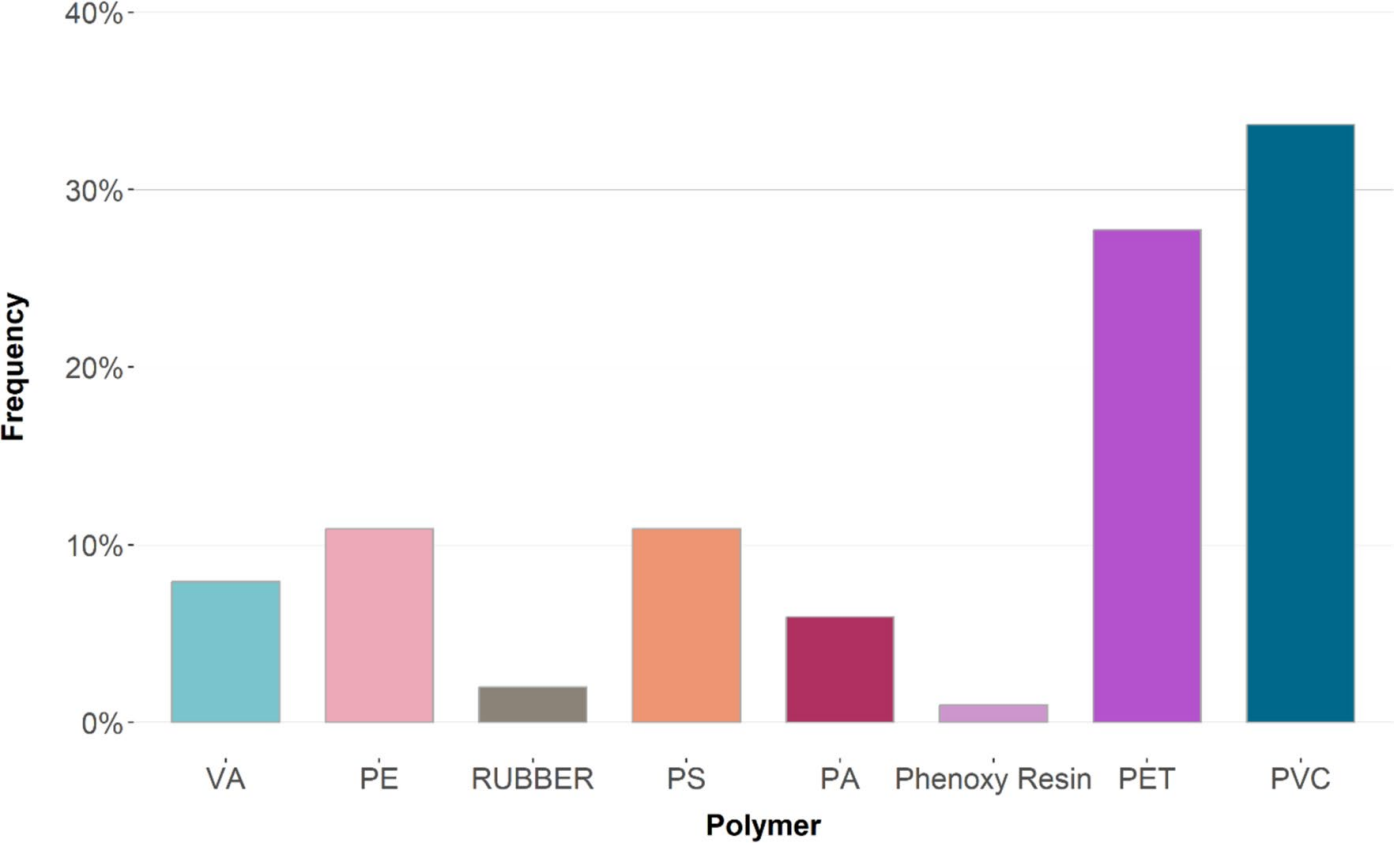
Frequency distribution of polymer types in sediments across all depths

For the µRaman spectroscopy analysis, a random subsample of 101 particles identified as microplastics was analysed for their polymer composition. The most common compositions found were polyvinyl chloride (PVC, 34%) and polyethylene terephthalate (PET, 28%). Other materials found include polyethylene (PE, 11%), polystyrene (PS, 11%), vinyl acetate (VA, 8%), polyamide (PA, 6%), rubber (2%) and phenoxy resin (1%) (Fig. 4). According to the total number of particles found, this result only accounts for 0.9% of the identified particles due to lacking resources. Thus, it has to be considered as not representative.

**Fig. 5:**
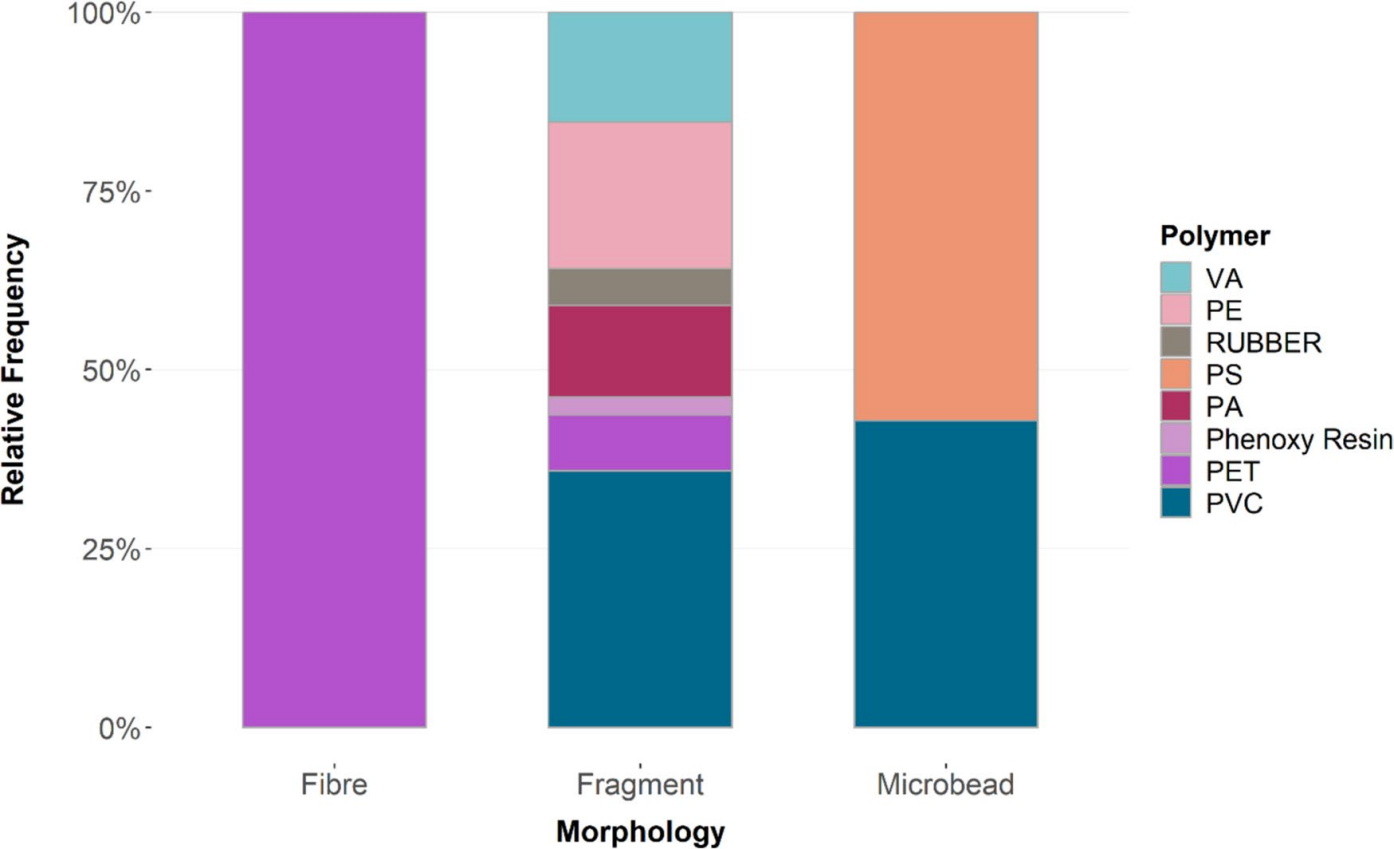
Frequency distribution of polymer types according to morphology in sediments across all depths

Assigned to particle morphologies, it is evident that all fibres are made of PET, microbeads consist of PS and PVC, while fragments show a variety of different polymer types (Fig. 5).

**Fig. 6:**
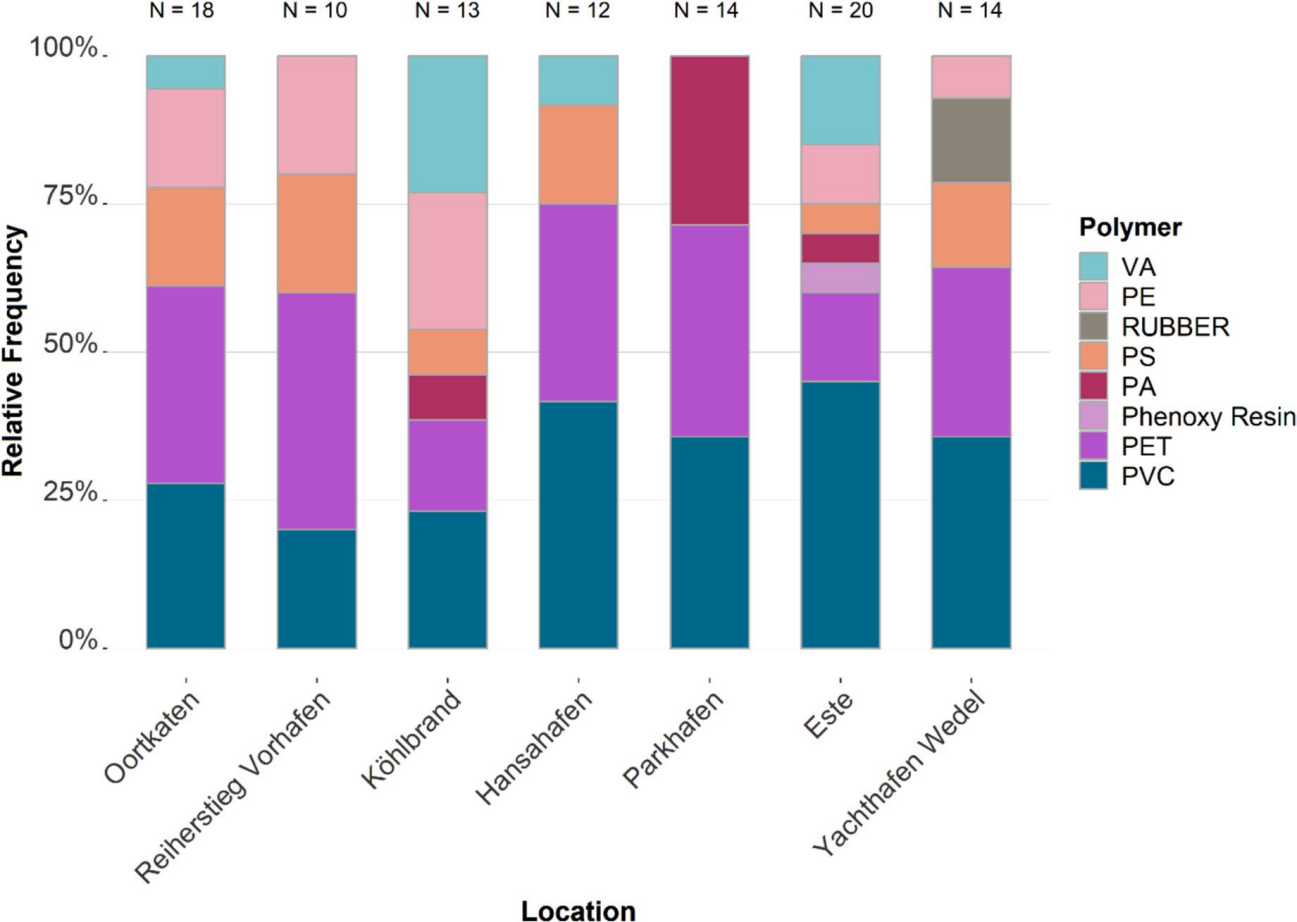
Frequency distribution of polymer composition in sediments across all depths according to location

At all locations, PVC and PET are present, as seen in Fig. 6. PS is present in almost every location except for Parkhafen. The location Außen-Este shows the highest variation in polymer types.

#### Sediment characteristics and water parameters

Organic matter (2 to 10%) and water content (30 to 103%) are considered to be high in all samples (SI6). However, a correlation according to Spearman between the microplastic concentration per kg dw and organic matter could not be detected (rho = 0.2, *p*-value > 0.05) whereas a highly significant positive correlation was found between microplastic content and water content (rho = 0.5, *p*-value < 0.05). A correlation between other taken water parameters could not be detected. Complete detected parameters are seen in Table SI4.

The grain size distribution analysis showed a high percentage of silt at all sites (SI2). Reiherstieg Vorhafen exhibits the highest clay quantity in almost all depth levels, except for the first one. The highest amount of sand can be found among all depth levels at the location Außen-Este (SI3).

A Spearman correlation analysis between microplastic particle concentration per kg dw and different grain size fractions (sand, coarse silt, medium silt and clay) revealed no correlations. However, a correlation taking into account particle morphology showed significant correlations between microbeads and coarse sand (rho 0.4, *p*-value < 0.05), microbeads and medium sand (rho 0.4, *p*-value < 0.05) and a highly significant correlation between microplastic concentration and fine sand (rho 0.6, *p*-value < 0.01).

## Discussion

### Spatial differences of microplastic concentration in the Hamburg port area

Microplastics were detected at all sampling sites and across all sediment depth levels analysed. High concentrations could be detected in every location, although the first depth level (approx. 0–2 cm bgl) across every site shows the lowest microplastic concentrations. This finding is in contrast to the assumption that concentration levels decrease with sediment depth as shown in a study from Zhou et al. (2021). Similar findings to the ones found in the depth levels of the port areas of Hamburg can be found in Ghayebzadeh et al. (2021). Here, 43.6% of microplastics were identified in the top 5 cm of sediments and 56.4% in the 5 to 15 cm in the Caspian Sea. The study explains that with the increase of permeability of sediments caused by microplastics, it is likely that more microplastics can be observed in the lower depths. Another reason could be due to the tidal influence in the river, which causes microplastic abundance fluctuations in intertidal sediments (Leads et al. 2023). Particles get repeatedly suspended, advected, deposited and resuspended by the tidally varying flows until they settle in regions of low flow velocity (Cheng et al. 2021). Due to the high sedimentation rates in port areas, suspended sediments settle within a few days and weeks in flow-calming zones (Hellmann et al. 1999). Meaning the sediment depth levels analysed in this study are sediments that are accumulated over short time periods. High concentrations of accumulated microplastics can therefore be held in place by rapidly deposited newer sediment, while the first layer is more dependent on flow rates and tidal influences. All samples were taken during a low tide; microplastic particles could possibly be in a resuspended state until they might settle and be held in place by the next accumulated sediment layer.

Oortkaten is the most upstream-located sampling site. Here, 317–6001 MP particles per kg dw were found. The highest concentration of 6001 was detected in the fourth sediment depth layer (approx. 30–50 cm bgl). The sampling site was located near a jetty with a few private boats that is a likely local source. Locations with higher human activities are more often found to be higher in MP concentration as seen in studies from, i.e. Eo et al. (2019), Jiang et al. (2019) and Khan et al. (2022). Another factor for high microplastic numbers could be that sampling sites were chosen by their similar sediment accumulation rates; thus, more microplastic particles are able to settle faster. This is in accordance with a study on the spatial distribution of microplastics by Mathalon and Hill (2014) that also found higher accumulation at downwind sites and in areas with reduced water flow.

The highest microplastic concentrations were detected at the location Reiherstieg Vorhafen (473–21,799 particles per kg dw). Here, the highest concentration can be observed in the third depth level (approx. 20–40 cm bgl). This site is characterised by logistic industries lining up around the riverbank and is located further upstream the river. It can be assumed that the industrial proximity might be of importance to these high rates. An increase of microplastic particles in urbanised areas and heavy-traffic roads could also be detected by Rimondi et al. (2022). Comparable to Oortkaten, this location was also chosen for its lower flow velocity, thus having a similar accumulation probability.

Further downstream the Elbe river lies the location Köhlbrand. The sampling took place near the Köhlbrand bridge, which is highly trafficked. Here, 2791–6236 MP per kg dw were detected with the fourth sediment depth level (approx. 15–30 cm bgl) carrying the highest contamination load. MP concentrations found were similar to the ones at Oortkaten. Other than the locations discussed above, this location was chosen out of interest, due to its high anthropogenic frequency. Its proximity to urban sources and heavy-traffic roads might also explain its high MP concentrations (Rimondi et al. 2022). A connection between the microplastic concentration in the sediment and the Köhlbrandhöft sewage treatment plant located in the area could not be established. Almost 90% of Hamburg’s wastewater enters the sewage treatment plant (Ratter 1999). The lack of a discernible connection could be due to MP being retained in sewage sludge and entering the environment via discharge as fertiliser (Hamann 2019). At Köhlbrand, fibres were found to be the third most abundant group of particles (1189 MP per kg dw). All of the fibres analysed at this site were in fact PET. PET fibres are typical for wash effluent according to Matsuguma et al. (2017); however, comparing the amount of fibres found at Köhlbrand to the other locations, it is visible that sites like Reiherstieg Vorhafen and Yachthafen Wedel have a much higher fibre concentration (Table 2). Results found here are therefore not visibly enhanced by the sewage treatment plant proximity. To ensure water depth in the waterways of Hamburg like Köhlbrand, dredging of sediment is frequently done (Hochfeld and Röper 2019). Due to this dredging, the sediment samples investigated are therefore freshly deposited suspended sediments that settled within a few days and weeks in current-calmed zones such as harbours (Hellmann et al. 1999).

The lowest MP concentration was detected at Hansahafen. The sampling site is located near a pier. In total, 60–526 MP per kg dw could be found, of which the highest concentration accumulated in the third depth layer (approx. 20–40 cm bgl). Compared to the other locations, concentrations are drastically lower. A possible explanation for this might be its branched, semi-enclosed location within the estuary. MP particles might be settling in different locations with a slowed flow velocity upon entry into this part of the estuary. Despite its proximity to the logistic industry, concentrations are low. This finding indicates that not only anthropogenic proximity plays a role in MP concentrations at certain locations but also river morphology (Gerolin et al. 2020). Permanent interference of river streams and sea waves in the estuaries causes the gradual accumulation of sediment load in the estuaries of the rivers (Ghayebzadeh et al. 2021). This might cause some areas of rivers to be less contaminated than others. Another interesting result was that Hansahafen has the lowest concentration of fragments (51 MP per kg dw) and fibres (114 MP per kg dw) found across all sampling locations. However, microbeads are the most abundant morphology with 1011 MP per kg dw (Table 2). A possible explanation might be the pier location, since microbeads are often also used as abrasive media in air-jet boat cleaning (Mathalon and Hill 2014, Sundt et al. 2014).

The next sampling location was Parkhafen, which is located near a big container terminal. Here, 2239–12,796 MP per kg dw were identified with the highest concentration being in the third layer (approx. 20–40 cm bgl). MP concentrations found here are the second-highest results in this study. Because of its closeness to the container terminal, high concentrations may result from the industrialised area (Rimondi et al. 2022). Harbours are semi-enclosed systems, which means microplastics can have a longer accumulation time with respect to open waters (Tesán Onrubia et al. 2021). Because of areas in the harbour basins with increased deposition, an accumulation of microplastics within the terminal might be a possible explanation for the high concentrations found in Parkhafen. Higher concentrations in areas of container terminals can be found in the study of Tesán Onrubia et al. (2021) who found 5576 to 379,965 MP km^−2^ in surface waters of North-Western Mediterranean harbours. Compared to the other locations analysed in that study, the Genova site which was in proximity to a container terminal had the highest amounts of MP.

The sampling site Außen-Este is located near the Este estuary, a tributary flowing into the upper tidal Elbe river. This site was lined with industry along the near riverbank. Across the river on the other side of the riverbank, the popular beach spot and residential area “Blankenese” is located. In total, 3687–4975 MP per kg dw could be extracted. The highest contamination was detected at the second depth level (approx. 2–20 cm bgl). Similar to the site Köhlbrand, this location was chosen out of interest to see if the receiving river has any influence on the microplastic abundance. Compared to the other results, this site however does not show any abnormalities, and therefore, a connection to the tributary is probably unlikely. Considering that the Este flows through the midsize city Buxtehude and no other considerable urban areas, a higher influx of MP into the Elbe is unlikely. The high contamination numbers could be linked to the location’s proximity to human and industrial activities (Rimondi et al. 2022).

The last sampling station was Yachthafen Wedel. As the name suggests, this station is located inside the yacht marina. Concentrations of 4485–7452 MP per kg dw were detected at this site. The highest concentration could be found at the depth level three (approx. 30–50 cm bgl). High concentrations can be explained by the proximity of anthropological impact as well as a slowed flow velocity caused by the bay-like structure of the marina. Naidoo et al. (2015) found harbour sediments and Yacht club areas to be highly contaminated with microplastics. Their study identified 745.4 ± 129.7 Mp per 500 ml in the sediment of five urban estuaries of KwaZulu-Natal (South Africa). This is much lower than in the study at hand.

Despite the slight local differences in microplastic concentration between sampling stations, the sediment in the Hamburg port area demonstrates high microplastic pollution rates. There are yet only a few studies with comparable high numbers, located in Asia (i.e. Choong et al. 2021, Eo et al. 2019, Manalu et al. 2017, Matsuguma et al. 2017), North America (i.e. Cheng et al. 2021), South America (i.e. Gerolin et al. 2020) and North-Western Mediterranean harbours (Tesán Onrubia et al. 2021). Microplastic studies in sediment from larger European harbours however are rarely reported. The study provided by Scherer et al. (2020) on the river Elbe reported much higher pollution rates as the results presented in this work with 3,350,000 ± 6,600,000 MP per m^3^. In this study, water samples were also taken, and on average, sediment samples had 600,000-fold higher MP concentrations compared to water samples. Interestingly enough, it is stated that the middle Elbe river shows much higher concentrations than the upper tidal Elbe river. They allege that MPs are likely to be separated by the Geesthacht barrage, and since the upper tidal Elbe river after the barrage is influenced by the tide, it prevents large accumulations of MP in sediment. Instead, the tidal activity would increase the transport of MPs into the North Sea because of the constant exchange of water bodies (Scherer et al. 2020). This would explain the concentration differences in the Elbe found by Scherer et al. (2020) and the current study. Being a major tributary to the North Sea and considering the overall high pollution rates of the river Elbe, further intensified studies with regard to spatial and temporal representativeness should be conducted, and also, the integration of the river Elbe within a potential monitoring programme should be striven for.

### Microplastic particle characteristics in the Hamburg port areas

The MP size distribution from all samples shows that MP frequency increases in the smallest size range (SI1). Other studies have also observed that the occurrence of small MPs in sediments is larger than that of large MPs (Choong et al. 2021; Ghayebzadeh et al. 2021; Klein et al. 2015; Leads et al. 2023; Utami et al. 2021). These small-sized microplastics pose a high potential threat towards aquatic life, since it increases the probability to be swallowed and ingested by aquatic animals (Ghayebzadeh et al. 2021; Liong et al. 2021).

For fragments, sizes ranged from 20 to 2126 µm. The sampling site with the largest fragments found with a median of 80 µm was Außen-Este and the smallest fragments with a median of 46 µm was Hansahafen. However, Hansahafen also had the lowest fragment concentration, which influences this result. Microbeads ranged between 20 and 617 µm; the largest ones with a median of 60 µm were again found at the site Außen-Este and the smallest with a median of 36 µm at Hansahafen. For fibres, the sizes ranged from 37 to 4899 µm with the largest fibres with a median of 679 µm found at Yachthafen Wedel and the smallest with a median of 323 µm at Parkhafen. For fragments and microbeads, Hansahafen samples had more small particles while more large ones can be found at Außen-Este. Size distributions at these locations may be attributed to their local differences. Hansahafen has the lowest MP contamination which alters the results. Larger particles found at Außen-Este might be due to the different sources of input around the riverbank.

Except for Hansahafen, fragments were the most common shape that could be detected. This is in accordance with many other studies that analysed MP in sediments (i.e. Choong et al. 2021; Eo et al. 2019; Khan et al. 2022; Leads et al. 2023; Liong et al. 2021; Mistri et al. 2021; Zhou et al. 2021). At Hansahafen, microbeads were the dominant particle morphology which is in accordance with the study from Scherer et al. (2020). Fibres were the least common particle morphology found in sediments of the Hamburg port area; however, several studies found them to be the most common morphology (i.e. Deng et al. 2020; Ghayebzadeh et al. 2021; Jiang et al. 2019; Rimondi et al. 2022, Utami et al. 2021). Scherer et al. (2020) state that reduced particle size and high proportions of fragments in the sediments indicate MP settling and fragmentation in the sediment. The lack of fibres in the sediments can be explained by the low settling velocities and constant water flow along the river, which potentially prevents fibres from settling. This would result in an increased abundance of fibres in the water phase and reduced accumulation in sediments (Scherer et al. 2020). Additionally, MP morphologies have been shown to influence the settling behaviour of particles with spherical microbeads, fragments or foams settling faster than elongated particles like fibres (Choong et al. 2021; Laursen et al. 2022).

Samples from sediments show a wider variety of polymer types than water (Scherer et al. 2020; Eo et al. 2019). The location with the most polymer diversity was Außen-Este while the least diversity can be found in Parkhafen. The lack of any popular commercially used plastic like PE in Parkhafen could be due to the fact that the location is purely for logistical and industrial usage. Therefore, contamination due to popular plastic garbage material that might derive from human proximity might not appear as frequent as in locations that might be used for tourism or leisure.

Other than polymers with a higher density than water like for example PS, PA, PET and PVC, oftentimes there can also be found particles like PE with a lower density (Barboza et al. 2019). Positively buoyant MPs may undergo processes like biofouling, weathering, inclusion of substances during production, formation of heteroaggregates or sorption of biomolecules which can increase particle density, causing them to settle (Laursen et al. 2022, Mathalon and Hill 2014, Matsuguma et al. 2017, Nizzetto et al. 2016, Scherer et al. 2020). The lack of PVC found by many studies might be explained through methodological differences. The separation of polymer types from the sample substrate is highly dependent on the density of the solution used. Therefore, studies applying densities below 1.40 g cm^−3^ are not capable to separate PVC with a density of 1.37–1.39 g cm^−3^ from the sediment samples (i.e. Blair et al. 2019, Ghayebzadeh et al. 2021, Jiwarungrueangkul et al. 2021, Leads et al. 2023, Naji et al. 2017). With added density from processes like biofouling, it becomes even more difficult to separate PVC, which is why higher densities for the solutions are recommended.

Another interesting aspect is the abundance of microbeads detected in this study. Microbeads were identified in every sample, a finding that is supported by several other studies on sediments (Claessens et al. 2011, Jiang et al. 2019, Klein et al. 2015, Mani et al. 2019, Scherer et al. 2020, Zhou et al. 2021). Microbeads pose an ecological danger due to their smaller surface area-to-volume ratio resulting in a slower photo-degradation. Therefore, the spherical microbeads stay longer in the environment compared to fragments and other morphologies of MPs (Torrance et al. 2021). These microbeads are typically found to be PS, meaning the popular claim, microbeads derive from cosmetic sources does not apply (Rochman et al. 2015; Torrance et al. 2021). These, usually made from PE or PP, are much more believed to be found in the water column due to their low density (Hidalgo-Ruz et al. 2012). PS and PVC microbeads on the other hand sink due to their higher density. It can be assumed that these microbeads have an industrial source of origin. Their applications range from cleaning products and printer toners to industrial products such as abrasive media (i.e. air-jet boat cleaning, oil and gas exploration production, textile printing and automotive manufacturing). Likewise, they are used as anti-slip and anti-blocking applications, as well as in the medical field (Sundt et. al. 2014). As for PS microbeads in specific, they are also frequently used as ion-exchange resin beads (Scherer et al. 2020). A study by Mani et al. (2019) states that these PS microbeads found within the river sediments are most likely polystyrene divinylbenzene (PS-DVB) ion-exchange resin (IER) beads. This claim was supported by an analysis of their chemical properties, comparisons with the manufacturer’s descriptions and visual characteristics of the beads. PS(-DVB) microbeads are commonly applied in water softening and various industrial purification processes, as well as in analytical laboratories, medicine, the food industry, horticulture and (waste-) water treatment. Additionally, the study by Scherer et al. (2020) concludes that PS-DVB microbeads potentially originate from the industrial areas of Dessau or Bitterfeld, where plastic processing industry is located. In these locations, they identified microbead concentration peaks indicating industrial emissions as a major source for local MP hotspots. 

**Fig. 7:**
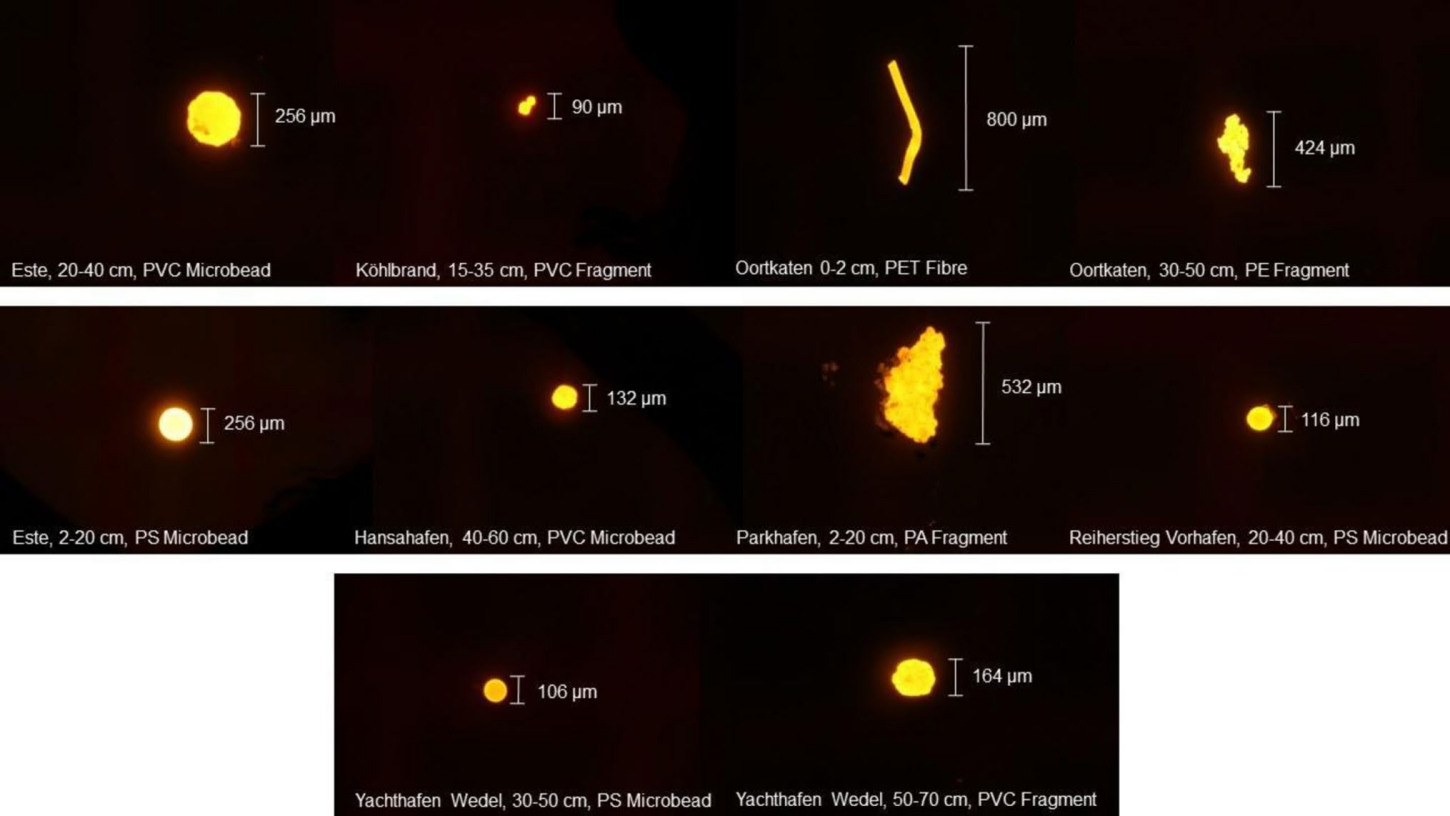
Microplastic particle comparison under the fluorescence microscopy

PVC microbeads are still not documented in any publications. Klein et al. (2015) allege that fragments may be polished to a round form by physical forces and therefore contribute to the number of spheres. As Fig. 7 depicts, PS and PVC microbeads display morphological differences supporting the theory posed by Klein et al. (2015). But comparing the PVC and PS microbeads in Fig. 7, the two round spheres do not show distinct morphological differences, except the signs of degradation on the surfaces of the PVC bead. These rough edges and degradation signs are also observed in Fig. 7. PVC is used for ship cleaning via air-jet; however, due to the morphological differences of all microbeads, multiple sources of origin seem probable.

### Correlations between microplastic particles and sediment characteristics

A correlation between sediment grain sizes and overall microplastic concentrations could not be found in this study. This is in accordance with studies like Deng et al. (2020); however, other studies state that sediments are seen to affect microplastics. The study from Marques Mendes et al. (2021) for example determined that fine-grain sediments such as mud accumulate higher MP concentrations than coarse sediments. They also observed that MP concentrations are greater in intertidal sediments because finer-grained sediments entrap more microplastics than coarser-grained sediments. In addition, a study from Enders et al. (2019) from benthic sediment of the Warnow Estuary (Germany) discovered a positive correlation between the concentration of low-density MP and fine-grained sediments. This occurrence is believed to be caused by flocculation processes; positively buoyant MP particles in estuarine environments are believed to form flocs with suspended fine-grain sediment and other suspended particles. This causes them to settle as soon as the hydrodynamics reduces (Laursen et al. 2022).

The significant correlation between microplastic concentrations and water content of the sediment is a factor of the grain size dominance of sand fractions. Both (water content and sand grain size) indicate that microplastic concentrations within the investigated size ranges are more affected by, e.g. velocities and sedimentation processes whereas organic matter within the study site is more likely to be due to local effects such as site-specific sources or primary production.

However, no correlation was found between grain sizes and overall MP; there is a correlation between microbeads and the sand fraction. In particular this is evident with coarse sand (rho 0.4, *p*-value < 0.05), medium sand (rho 0.4, *p*-value < 0.05) and even more distinct fine sand (rho 0.6, *p*-value < 0.01). It is striking that microbeads appear in the same size fractions (20–617 µm) as coarse (630 µm), medium (200 µm) and fine (63 µm) sand. The results may be interrelated with the morphology of microbeads (spherular) which are therefore similar to the distributions of grain sizes based on STOKE’s law (DIN ISO 11277). This finding has yet not been indicated in any other study, and intensified studies need to be conducted to understand and describe the movement of microbeads within sediments even though a grain size distribution not yet sufficiently explains the accumulation and distribution of fragments and fibres.

## Conclusion

In conclusion, microplastic contamination could be detected at all locations at a high quantity. Above all, the locations within the port (Reiherstieg Vorhafen, Parkhafen) show elevated microplastic concentrations. Fragments were the most abundant particle morphology, though it is especially concerning that large quantities of microbeads could be found at every location. PVC was the most frequent polymer type from all particles analysed. In this study, a correlation between microplastics and grain size could not be detected; however, a correlation between microbeads and the sand fraction could be identified. Our study shows that harbour environments may pose an important sink for microplastic contamination as conditions within harbour basins enhance MP deposition. To fully grasp deposition processes and the relation to MP sources and sinks in rivers, it is necessary to further gain an understanding of the related microplastic abundances in the water column as well as to analyse other important hydrological factors in future studies.

## Supplementary Information

Below is the link to the electronic supplementary material.

Supplementary file1 (DOCX 722 KB)

## Data Availability

Data will be made available on request.
