# Supplementary material for: Microplastics in the Hamburg port area—an analysis of sediment depth profiles along the upper Elbe river, Germany
Source: Environ Sci Pollut Res Int. 2025 Feb 1;32(8):4825–40. doi: 10.1007/s11356-025-35972-w (PMC11850550; doi:10.1007/s11356-025-35972-w)
Supplement: Supplementary file 1 — Supplementary file1 (DOCX 722 KB) [file 11356_2025_35972_MOESM1_ESM.docx]

**Supplementary to Motyl and Fischer 2024: Microplastics in the Hamburg port area – An analysis of sediment depth profiles along the upper Elbe river, Germany**


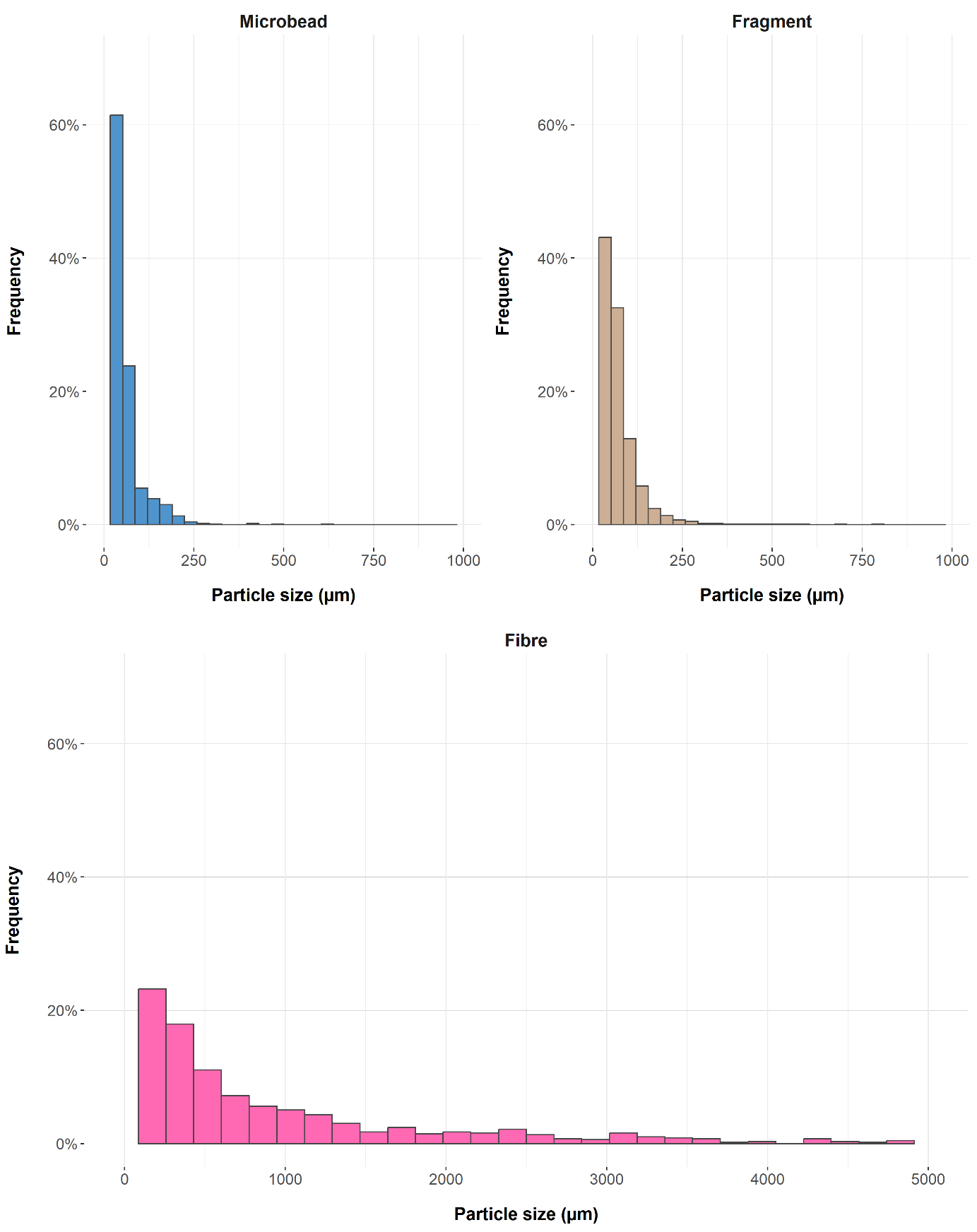


**SI 1** Frequency distribution of particle sizes (μm) of microbeads, fragments and fibres


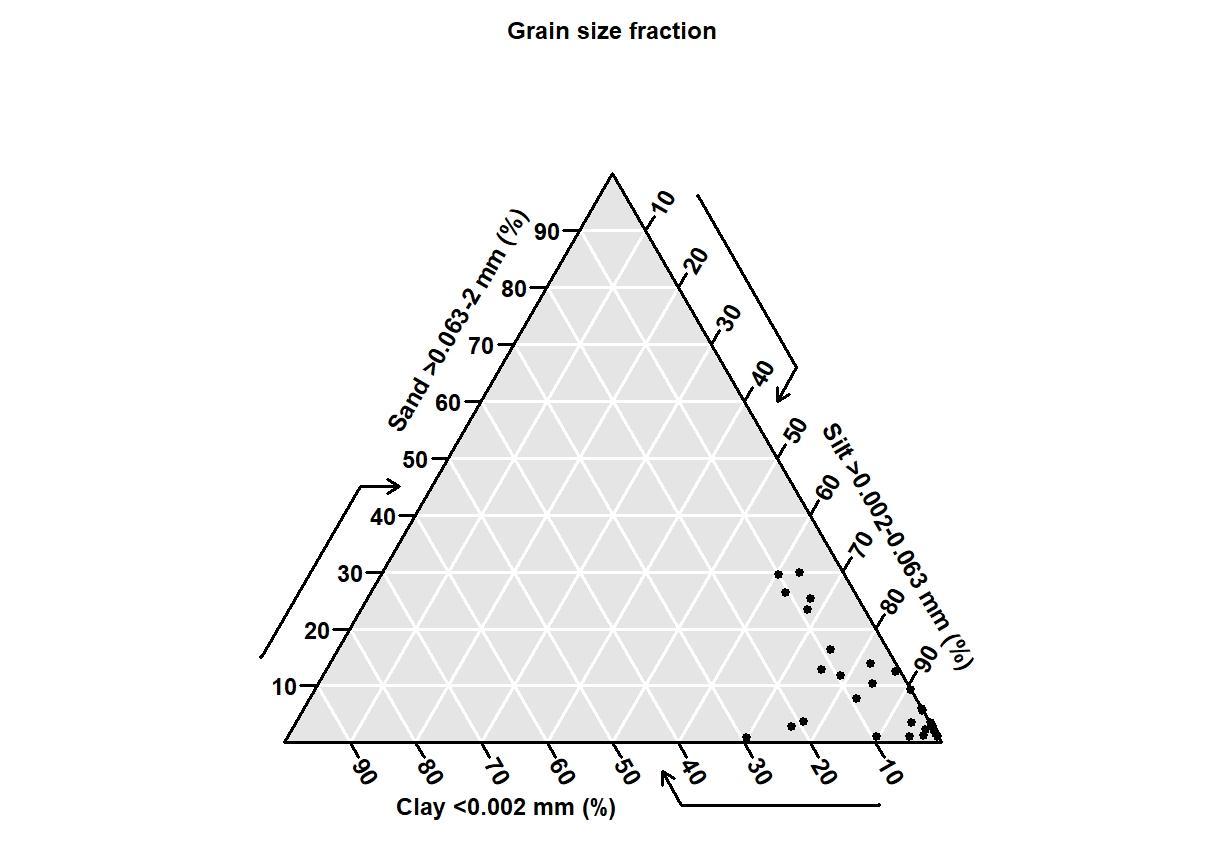


**SI 2** Triangular diagram of the grain size classes

*
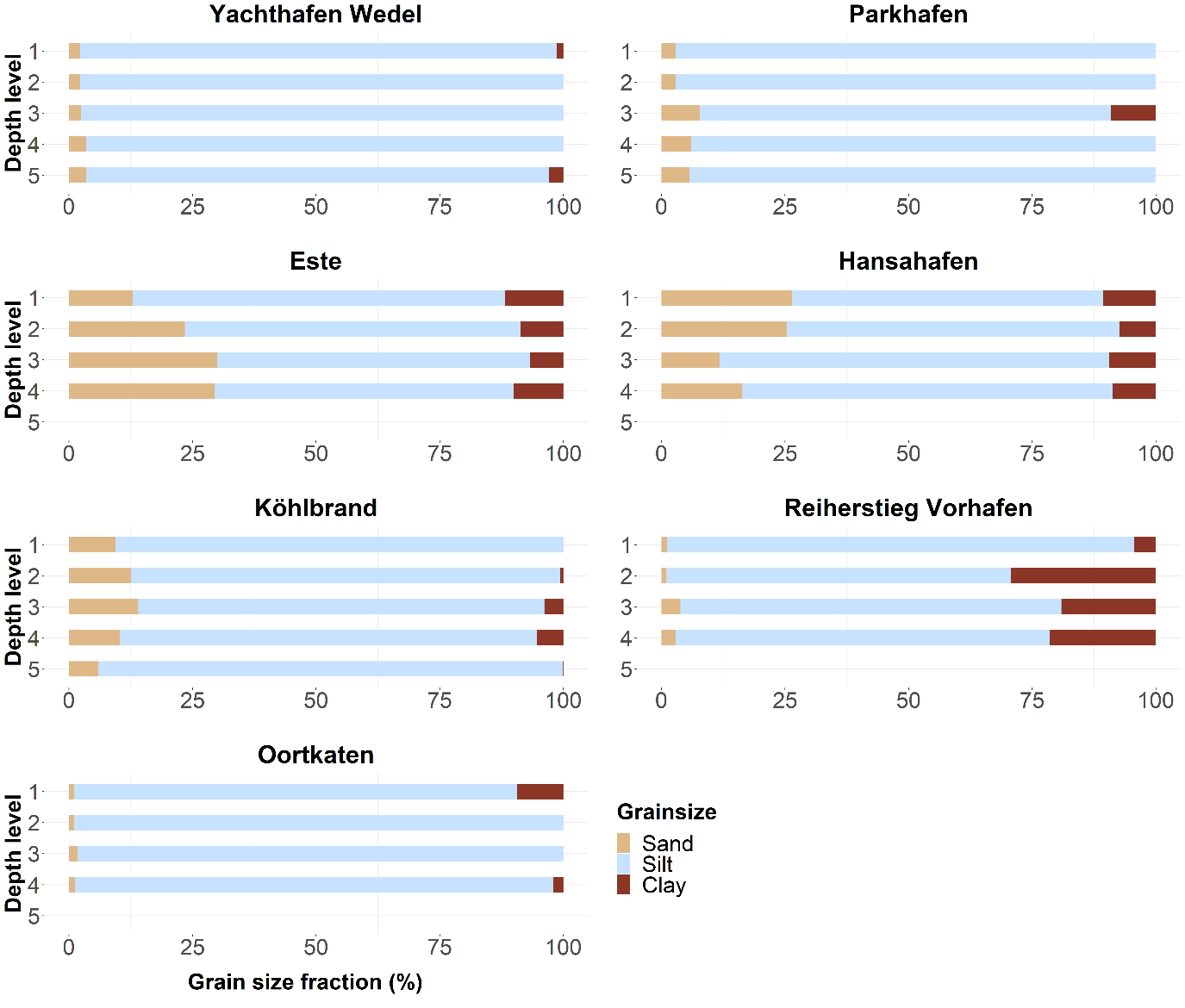
*

**SI 3** Grain size fractions in % of sand, silt and clay among all locations and depth levels

| **Location** | **Alti (m)** | **P-ext (dbar)** | **Press (dbar)** | **Temp (°C)** | **Cond (mS/cm)** | **pH** | **Chl_A (µg/l)** | **Turb (FTU)** | **sat (%)** | **DOmgS (mg/l)** | **DOmlS (ml/l)** | **TiltX (°)** | **TiltY (°)** | **CAP25 (mS/cm)** | **SALIN (PSU)** | **SIGMA (kg/m3)** | | **SOUND (m/s)** | **VCSP (m/s)** | **VDIR (°N)** |
| --- | --- | --- | --- | --- | --- | --- | --- | --- | --- | --- | --- | --- | --- | --- | --- | --- | --- | --- | --- | --- |
| Oortkaten | 7 | 1 | 1 | 16 | 0.7 | 9 | 12 | 89 | 63 | 6 | 4 | -5 | 4 | 0.8 | 0,4 | -0,8 | 1471 | | 0,5 | 127 |
| Reiherstieg Vorhafen | 7 | 2 | 3 | 17 | 1.2 | 8 | 11 | 93 | 64 | 6 | 4 | -4 | 5 | 1.4 | 0,7 | -0,8 | 1475 | | 0,4 | 139 |
| Köhlbrand | 8 | 7 | 8 | 17 | 0.9 | 8 | 6 | 147 | 60 | 6 | 4 | -6 | 7 | 1.1 | 0,5 | -0,8 | 1474 | | 0,2 | 173 |
| Hansahafen | 8 | 5 | 6 | 18 | 1.1 | 8 | 9 | 257 | 51 | 5 | 3 | -3 | 5 | 1.3 | 0,6 | -0,9 | 1477 | | 0,3 | 192 |
| Parkhafen | 8 | 8 | 8 | 17 | 1.0 | 8 | 4 | 247 | 53 | 5 | 4 | -1 | 2 | 1.2 | 0,6 | -0,8 | 1475 | | 0,2 | 172 |
| Este | 6 | 1 | 2 | 17 | 0.7 | 9 | 2 | 210 | 62 | 6 | 4 | -10 | 6 | 0.9 | 0,4 | -0,8 | 1472 | | 0,4 | 127 |
| Yachthafen Wedel | 6 | 2 | 2 | 17 | 1.1 | 8 | 3 | 177 | 56 | 5 | 4 | -4 | 15 | 1.4 | 0,7 | -0,7 | 1474 | | 0,5 | 141 |

**SI 4** Mean values of the CTD data taken at the sample locations

| **Location** | **Depth level** | **Depth bgl (cm)** | **Correction factor (per 1 kg dry weight)** | **Fragments** | | | **Microbeads** | **Fiber** | **Particles in total** |
| --- | --- | --- | --- | --- | --- | --- | --- | --- | --- |
| Oortkaten | 1 | 0-2 | 63.40 | | 63 | 254 | | 0 | 317 |
| Oortkaten | 2 | 2-10 | 27.42 | | 274 | 1344 | | 137 | 1755 |
| Oortkaten | 3 | 10-30 | 17.73 | | 18 | 1596 | | 18 | 1631 |
| Oortkaten | 4 | 30-50 | 14.46 | | 4381 | 1258 | | 361 | 6001 |
| Reiherstieg Vorhafen | 1 | 0-2 | 29.58 | | 0 | 473 | | 0 | 473 |
| Reiherstieg Vorhafen | 2 | 2-20 | 13.15 | | 11466 | 1078 | | 1052 | 13596 |
| Reiherstieg Vorhafen | 3 | 20-40 | 17.12 | | 17535 | 1575 | | 2688 | 21799 |
| Reiherstieg Vorhafen | 4 | 40-60 | 16.97 | | 11252 | 1154 | | 1222 | 13628 |
| Köhlbrand | 1 | 0-2 | 18.36 | | 2424 | 239 | | 129 | 2791 |
| Köhlbrand | 2 | 2-15 | 19.39 | | 5254 | 426 | | 388 | 6068 |
| Köhlbrand | 3 | 15-35 | 17.00 | | 6408 | 272 | | 204 | 6884 |
| Köhlbrand | 4 | 35-55 | 18.64 | | 6673 | 746 | | 335 | 7754 |
| Köhlbrand | 5 | 55-70 | 16.63 | | 5738 | 366 | | 133 | 6236 |
| Hansahafen | 1 | 0-2 | 19,98 | | 0 | 60 | | 0 | 60 |
| Hansahafen | 2 | 2-20 | 16,31 | | 0 | 130 | | 114 | 245 |

| Hansahafen | 3 | 20-40 | 9,93 | 30 | 497 | 0 | 526 |
| --- | --- | --- | --- | --- | --- | --- | --- |
| Hansahafen | 4 | 40-60 | 10,47 | 21 | 324 | 0 | 345 |
| Parkhafen | 1 | 0-2 | 27,99 | 1903 | 168 | 168 | 2239 |
| Parkhafen | 2 | 2-20 | 22,41 | 4661 | 291 | 426 | 5378 |
| Parkhafen | 3 | 20-40 | 16,86 | 11801 | 506 | 489 | 12796 |
| Parkhafen | 4 | 40-60 | 15,60 | 10923 | 702 | 343 | 11969 |
| Parkhafen | 5 | 60-80 | 20,89 | 9337 | 480 | 167 | 9984 |
| Este | 1 | 0-2 | 26,53 | 3289 | 212 | 186 | 3687 |
| Este | 2 | 2-20 | 19,68 | 6297 | 433 | 236 | 6966 |
| Este | 3 | 20-40 | 10,91 | 4353 | 240 | 382 | 4975 |
| Este | 4 | 40-60 | 12,79 | 6431 | 281 | 153 | 6866 |
| Yachthafen Wedel | 1 | 0-2 | 24,51 | 4093 | 98 | 294 | 4485 |
| Yachthafen Wedel | 2 | 2-30 | 13,85 | 6023 | 457 | 346 | 6826 |
| Yachthafen Wedel | 3 | 30-50 | 15,12 | 6560 | 438 | 453 | 7452 |
| Yachthafen Wedel | 4 | 50-70 | 15,10 | 5707 | 377 | 513 | 6598 |

| Yachthafen Wedel | 5 | 70-90 | 17,54 | 5787 | 561 | 614 | 6962 |
| --- | --- | --- | --- | --- | --- | --- | --- |

**SI 5** Microplastic concentrations per locations and depth levels in addition to their correction factor

| Pedological data | | | |  | | Grain size data | | | | | | | |
| --- | --- | --- | --- | --- | --- | --- | --- | --- | --- | --- | --- | --- | --- |
| **Location** | **Depth  level** | **Organic matter content (%)** | **Water  content (%)** | **fS (%)** | **mS (%)** | **gS (%)** | **gU (%)** | **mU (%)** | **fU (%)** | **T (%)** | | **Fine  fraction  (%)** | **Sand  fraction (%)** |
| Oortkaten | 1 | 10 | 92 | 0 | 2 | 0 | 42 | 29 | 12 | 15 | 98 | | 2 |
| Oortkaten | 2 | 10 | 86 | 2 | 0 | 1 | 79 | 12 | 5 | 1 | 97 | | 3 |
| Oortkaten | 3 | 10 | 81 | 2 | 0 | 1 | 74 | 20 | 4 | -1 | 97 | | 3 |
| Oortkaten | 4 | 10 | 80 | 1 | 0 | 0 | 78 | 15 | 6 | 1 | 99 | | 1 |
| Reiherstieg Vorhafen | 1 | 9 | 88 | 1 | 0 | 0 | 57 | 23 | 10 | 9 | 98 | | 2 |
| Reiherstieg Vorhafen | 2 | 10 | 75 | 1 | 0 | 0 | 87 | 2 | 5 | 4 | 98 | | 2 |
| Reiherstieg Vorhafen | 3 | 10 | 74 | 2 | 0 | 1 | 89 | 3 | 4 | 1 | 97 | | 3 |
| Reiherstieg Vorhafen | 4 | 9 | 74 | 1 | 0 | 1 | 87 | 3 | 4 | 3 | 97 | | 3 |
| Köhlbrand | 1 | 6 | 80 | 11 | 1 | 0 | 60 | 11 | 7 | 10 | 88 | | 12 |
| Köhlbrand | 2 | 5 | 65 | 15 | 0 | 0 | 73 | 5 | 3 | 4 | 85 | | 15 |
| Köhlbrand | 3 | 5 | 67 | 16 | 0 | 0 | 74 | 4 | 3 | 2 | 83 | | 17 |
| Köhlbrand | 4 | 6 | 103 | 14 | 0 | 1 | 76 | 4 | 2 | 3 | 85 | | 15 |
| Köhlbrand | 5 | 6 | 30 | 9 | 1 | 1 | 81 | 4 | 3 | 2 | 90 | | 10 |
| Hansahafen | 1 | 6 | 87 | 32 | 1 | 0 | 49 | 5 | 4 | 9 | 67 | | 33 |
| Hansahafen | 2 | 5 | 71 | 29 | 1 | 0 | 67 | 0 | 1 | 1 | 70 | | 30 |
| Hansahafen | 3 | 5 | 65 | 17 | 0 | 0 | 83 | -1 | 1 | 0 | 83 | | 17 |
| Hansahafen | 4 | 5 | 63 | 20 | 0 | 0 | 65 | 5 | 3 | 6 | 80 | | 20 |
| Hansahafen | 5 | 6 | 87 | 32 | 1 | 0 | 49 | 5 | 4 | 9 | 67 | | 33 |
| Parkhafen | 1 | 7 | 52 | 3 | 1 | 0 | 53 | 25 | 10 | 8 | 96 | | 4 |
| Parkhafen | 2 | 7 | 84 | 3 | 0 | 0 | 72 | 13 | 6 | 5 | 96 | | 4 |
| Parkhafen | 3 | 7 | 69 | 3 | 0 | 0 | 17 | 24 | 18 | 37 | 96 | | 4 |
| Parkhafen | 4 | 7 | 68 | 3 | 7 | 0 | 80 | 3 | 3 | 3 | 89 | | 11 |
| Parkhafen | 5 | 7 | 102 | 6 | 1 | 0 | 31 | 31 | 16 | 15 | 93 | | 7 |
| Este | 1 | 5 | 74 | 11 | 0 | 0 | 68 | 6 | 5 | 8 | 88 | | 12 |
| Este | 2 | 3 | 55 | 41 | 0 | 0 | 38 | 9 | 8 | 4 | 58 | | 42 |
| Este | 3 | 2 | 39 | 37 | 0 | 0 | 63 | 1 | 1 | -2 | 63 | | 37 |
| Este | 4 | 3 | 45 | 40 | 1 | 0 | 60 | 2 | 1 | -3 | 59 | | 41 |
| Yachthafen Wedel | 1 | 6 | 75 | 2 | 0 | 0 | 61 | 31 | 4 | 3 | 98 | | 2 |
| Yachthafen Wedel | 2 | 7 | 74 | 2 | 0 | 0 | 95 | 2 | 2 | -1 | 98 | | 2 |
| Yachthafen Wedel | 3 | 6 | 75 | 2 | 0 | 0 | 97 | 2 | 1 | -3 | 98 | | 2 |
| Yachthafen Wedel | 4 | 7 | 74 | 2 | 0 | 0 | 97 | 1 | 1 | -1 | 97 | | 3 |
| Yachthafen Wedel | 5 | 7 | 73 | 2 | 1 | 1 | 97 | 1 | 1 | -2 | 97 | | 3 |

**SI 6** Grain size fraction, organic matter and water content in % per location and depth level
